# Oncological safety of autologous breast reconstruction after mastectomy for invasive breast cancer

**DOI:** 10.1186/s12885-018-4912-6

**Published:** 2018-10-19

**Authors:** Joachim Geers, Hans Wildiers, Katrien Van Calster, Annouschka Laenen, Giuseppe Floris, Marc Vandevoort, Gerd Fabre, Ines Nevelsteen, Ann Smeets

**Affiliations:** 1 Multidisciplinary Breast Centre, University Hospitals Leuven, Herestraat 49, 3000 Leuven, Belgium; 2 Department of General Medical Oncology, University Hospitals Leuven, Herestraat 49, 3000 Leuven, Belgium; 3 Department of Public Health and Primary Care, Interuniversity Institute of Biostatistics and Statistical Bioinformatics, University Hospitals Sint-Raphaël, Kapucijnenvoer 35, blok D, bus 7001, 3000 Leuven, Belgium; 4 Department of Imaging and Pathology, University Hospitals Leuven, Herestraat 49, 3000 Leuven, Belgium; 5 Department of Plastic, Reconstructive and Aesthetic Surgery, University Hospitals Leuven, Herestraat 49, 3000 Leuven, Belgium; 6 Department of Oncology, Surgical Oncology University Hospitals Leuven, Herestraat 49, 3000 Leuven, Belgium

**Keywords:** Autologous breast reconstruction, Invasive breast cancer, Tumour dormancy, Surgical stress, Metastases

## Abstract

**Background:**

The number of patients requesting autologous breast reconstruction (ABR) after mastectomy for breast cancer has increased over the past decades. However, concern has been expressed about the oncological safety of ABR. The aim of our study was to assess the effect of ABR on distant relapse.

**Methods:**

In this retrospective cohort study, data was analysed from patients who underwent mastectomy for invasive breast cancer in University Hospitals Leuven between 2000 and 2011. In total, 2326 consecutive patients were included, 485 who underwent mastectomy with ABR and 1841 who underwent mastectomy alone. The risk of relapse in both groups was calculated using a Cox proportional hazards analysis, adjusted for established prognostic factors. ABR was considered as a time-dependent variable. Additionally, the evolution of the risk over follow-up time was calculated.

**Results:**

With a median follow-up of 68 months, 8% of patients in the reconstruction group developed distant metastases compared to 15% in the mastectomy alone group (univariate HR 0.70, 95% CI 0.50–0.97, *p* = 0.0323). However, after adjustment for potential confounding factors in a Cox multivariable analysis, the risk of distant relapse was no longer significantly different between groups (multivariate HR 0.82, 95% CI 0.55–1.22, *p* = 0.3301). Moreover, the risk of metastasis after reconstruction was not time-dependent.

**Conclusions:**

These findings suggest that there is no effect of ABR on distant relapse rate and thus that ABR is an oncological safe procedure. The rate of local recurrence was too low to make any significant conclusions.

## Background

The number of patients requesting an autologous breast reconstruction (ABR) after mastectomy for breast cancer has increased over the past decades. An ABR with a perforator flap is considered a good option by many surgeons for such a reconstruction. It allows the transfer of the patient’s own skin and fat in a reliable manner with minimal donor site morbidity [1]. However, concerns have been raised about the oncological safety of an ABR, considering the significant amount of surgical stress of an ABR. Multiple studies have provided evidence on the concept of tumour dormancy in breast cancer patients [2–9]. Patients may harbour dormant micrometastases at the time of the ABR. This surgical trauma may activate these dormant micrometastases, resulting in early distant metastatic disease, a surgery-driven escape from dormancy [10–18]. Based on this hypothesis, Isern et al. noticed a higher risk of breast cancer recurrence in patients who had an ABR [19]. However, a more recent report of the same group was not able to confirm this increased risk of breast cancer recurrence for patients who underwent a delayed ABR [20].

The aim of this study was to evaluate the effect of the ABR on distant metastasis by evaluating the distant relapse rate in patients who underwent an ABR and those who underwent only a mastectomy. Additionally, the risk of metastasis after reconstruction was analysed whether it was time-dependent. If the reconstruction would provoke earlier appearance of metastases, an increase of metastases for earlier follow-up times compared to later follow-up times would be expected.

## Methods

### Study population

**Fig. 1:**
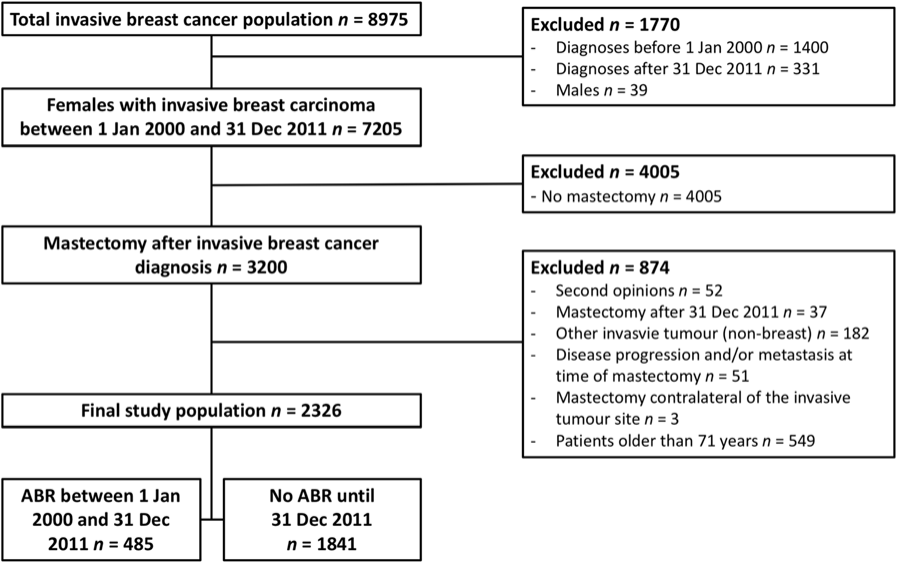
Flowchart of the study population

This retrospective study was approved by the Medical Ethical Committee of the University Hospitals Leuven (S54875). A prospectively maintained database was used to identify all female patients with invasive breast cancer who underwent mastectomy from January 2000 until December 2011. Most patients had a large portion of carcinoma in situ as well, making them eligible for a treatment by mastectomy. Patients with only a carcinoma in situ were excluded. A final study population of 2326 patients was achieved after applying in- & exclusion criteria as shown in Fig. 1. The age cut off was based on the oldest patient in the reconstruction group.

### Treatment

A team of 5 experienced breast surgeons performed the mastectomy. The ABR was carried out by 3 reconstructive plastic surgeons. In conjunction with the mastectomy, all patients underwent axillary staging by sentinel lymph node biopsy and/or axillary lymph node dissection. Isolated tumour cells alone are not considered as a positive lymph node status, whereas micrometastases are considered as lymph node positive. All patients received adjuvant treatment according to the institutional guidelines. The main indication for immediate ABR was early stage breast cancer with a low estimated risk for adjuvant radiotherapy and/or chemotherapy. Therefore, comparatively few immediate breast reconstructions were performed. Delayed reconstruction was offered to patients preferably at least 2 years after the primary surgery. Patients who opted for another reconstruction technique (e.g. implant based) are considered in the non-reconstruction group, as the amount of surgical stress is significantly lower with these techniques.

### Follow-up and outcome

The patients were all followed at the Multidisciplinary Breast Centre of the University Hospitals Leuven. The follow-up started from the time of mastectomy until the date of relapse, the date of death, or the date of last follow-up. As patients are followed at least annually at our centre, patients were considered lost to follow-up, if patients were still alive and the date of the last follow-up exam was more than 18 months before the end of the study. Local and axillary nodal recurrences were considered as locoregional recurrences. Metastases were considered as distant relapse. Contralateral invasive breast tumours were considered as new primary breast tumours. To test the hypothesis of tumour dormancy, the focus of this study was on the distant metastases.

### Statistical analysis

The analysis of the patient data was based on the Cox proportional hazards model. To account for the occurrence of a delayed ABR, the model included the ABR status as a time-varying covariate. Differences between both groups (ABR and non-ABR) were analysed. Variables presented with percentage were analysed using a Chi-square test and the variables summarized by medians and range were analysed using a Mann-Whitney U test. A multivariable model including several prognostic factors was used to correct for possible confounding. Age at mastectomy, tumour grade, invasive tumour size, tumour type, lymphovascular invasion (LVI), lymph node status, ER/PR status and Her-2 status were considered as possible confounders [21–23]. Follow-up summary statistics are based on the Kaplan-Meier estimates of potential follow-up [24]. To explore whether the development of metastases after an ABR was time-dependent, the cumulative probability to develop metastases was calculated over time (Kaplan-Meier estimates). Furthermore, a graph was constructed to present the evolution of the risk of metastasis over follow-up time. In this graph, the hazard (risk) of developing metastases was calculated within a (moving) time-window of 2 years to obtain a sufficiently smooth curve. The risk at a specific follow-up time was then calculated based on values observed in the period defined by the indicated time plus and minus 2 years. All statistical analyses were performed using SAS® software, version 9.2 (AS Institute Inc., Cary, NC, USA).

## Results

### Patient and tumour characteristics (Table 1)

A total of 2326 patients met the inclusion criteria, 485 patients underwent an ABR and 1841 patients underwent a mastectomy alone. An immediate reconstruction was performed in 143/485 patients (30%), a delayed reconstruction in 342/485 patients (70%). Overall, the time from mastectomy to delayed breast reconstruction ranged from 1 to 117 months and a median of 22 months. ABR was performed using a variety of microvascular flaps, including deep inferior epigastric perforator (DIEP) flap (*n* = 417), superficial inferior epigastric artery (SIEA) flap (*n* = 27), superior gluteal artery perforator (SGAP) flap (*n* = 19), transverse rectus abdominus myocutaneus (TRAM) flap (*n* = 2), or a combination of microvascular flaps (*n* = 20). Clinicopathological characteristics for the entire study population by reconstruction group are shown in Table 1. The median age at mastectomy, the median Nottingham Prognostic Index (NPI), the median invasive tumour size in mm and the lymph node status were significantly different between both groups (*p*-value < 0.05). The median follow-up time of the entire cohort (from mastectomy to last contact) was 68 months (range 1–153 months). The median follow-up time for the ABR group was 76 months (range 4–152 months) and 68 months for the non-ABR group (range 1–153 months). One hundred forty-three of 2124 alive patients were lost to follow-up (7%), 22 patients were lost to follow-up in the ABR-group (5%) and 121 patients in the non-ABR group.

**Table 1:** Comparison of patient- and tumour characteristics in the non-ABR and the ABR group

Variable	Non-ABR (*n* = 1841)		ABR (*n* = 485)		*p*-value
	*n*	%	*n*	%	
Age at mastectomy, median (range)	55 (23–71)		47 (24–71)		< 0.001*
- Missing data	0		0		
NPI, median (range)	4.6 (2.0–9.1)		4.5 (2.0–8.2)		0.007*
- Missing data	221		42		
Invasive tumour size in mm, median (range)	30 (0–180)		25 (0–160)		< 0.001*
- Missing data	105		18		
In situ tumour size in mm, median (range)	49 (0–170)		49 (0–170)		0.965
- Missing data	572		132		
Tumour grade					0.663
- 1	151	8	45	9	
- 2	816	45	206	43	
- 3	850	47	225	47	
- Missing data	24		9		
Lymph node status					0.020*
- Negative	739	47	229	54	
- Positive	817	53	196	46	
- Missing data	285		60		
Lymphovascular invasion					0.945
- No	872	67	222	67	
- Yes	432	33	109	33	
- Missing data	537		154		
Tumour type					0.290
- IDC	1360	74	372	77	
- ILC	325	18	71	15	
- Other	156	8	42	9	
- Missing data	0		0		
ER/PR					0.948
- Negative	389	21	103	22	
- Positive	1424	79	374	78	
- Missing data	28		8		
Her-2					0.892
- Negative	1419	81	375	80	
- Positive	342	19	92	20	
- Missing data	80		18		
Neoadjuvant treatment					
- Yes	275	15	56	12	0.057
- No	1566	85	429	88	
- Missing data	0		0		

Variables presented with percentages are analysed using a Chi-square test. Variables summarized by medians and range are analysed using a Mann-Whitney U test. All reported *p*-values are two-sided

*NPI* Nottingham Prognostic Index, *IDC* invasive ductal carcinoma, *ILC* invasive lobular carcinoma, *ER* oestrogen receptor, *PR* progesterone receptor, *Her-2* Human epidermal growth factor receptor

*denotes statistical significance (*p* < 0.05)

### Outcome

**Fig. 2:**
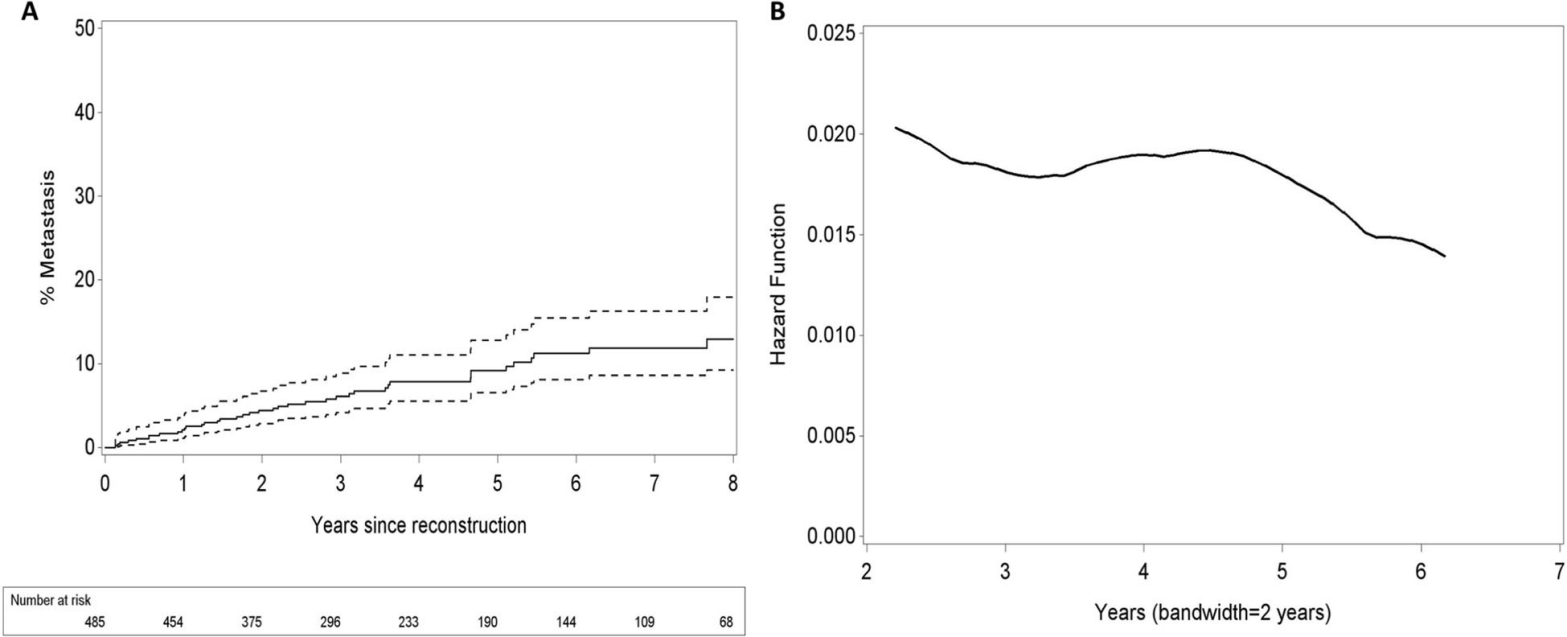
**a** Kaplan Meier estimates for the cumulative hazard of metastasis after an ABR. **b** Smoothed hazard function over follow-up time (in years). The risk at a specific follow-up time is based on values observed in the period defined by the indicated time +/− 2 years

Overall, 323/2326 (14%) patients developed distant metastases. 282/1841 (15%) patients in the mastectomy alone group and 41/485 (8%) patients in the ABR group had metastases (univariate HR 0.70, 95% CI 0.50–0.97, *p* = 0.0323). After adjustment for possible confounding, the multivariable Cox model showed no longer a statistically significant difference in distant relapse risk between patients with and without an ABR (multivariate HR 0.82, CI 0.55–1.22, *p* = 0.3301) (Table 2). Tumour grade and lymph node status were the most important prognostic factors (both *p* <  0.0001). As the variable LVI contained a high percentage of missing data, LVI was considered as a three-category variable with values ‘Yes’, No and Unknown, to avoid the loss of a large number of observations. Next, the development of metastases after ABR was analysed whether it was time-dependent. Kaplan Meier estimates for the cumulative hazard of metastases after reconstruction (Fig. 2a) were calculated. The results do not demonstrate an increase of metastases for earlier follow-up times compared to later follow-up times. Additionally, the smoothed hazard function suggests a rather stable risk up until 5 years after the ABR (Fig. 2b).

**Table 2:** Hazard ratios for metastasis from the multivariable Cox model

	Hazard Ratio	95% CI	*p*-value
ABR performed	0.82	[0.55–1.22]	0.3301
Age at mastectomy	1.00	[0.98–1.01]	0.5176
Tumour grade	2.56	[1.87–3.51]	<.0001*
Invasive tumour size (mm)	1.00	[1.00–1.01]	0.4547
Tumour type ILC	1.17	[0.75–1.82]	0.4920
Tumour type other	0.95	[0.54–1.65]	0.8505
LVI yes	1.66	[1.17–2.35]	0.0047*
LVI unknown	1.72	[1.21–2.44]	0.0026*
Lymph node status positive	2.13	[1.54–2.94]	<.0001*
ER/PR positive	0.62	[0.45–0.86]	0.0046*
Her-2 positive	0.77	[0.54–1.10]	0.1537

Factors: HR > 1 (< 1) means higher (lower) risk for patients in the indicated category than reference. Covariates: HR > 1 (< 1) means higher (lower) risk with increasing values of the covariate

*ABR* autologous breast reconstruction, *CI* confidence interval, *LVI* lymphovascular invasion, *ILC* invasive lobular carcinoma, *ER* oestrogen receptor, *PR* progesterone receptor, *Her-2* Human epidermal growth factor receptor

*denotes statistical significance (*p* < 0.05)

**Fig. 3:**
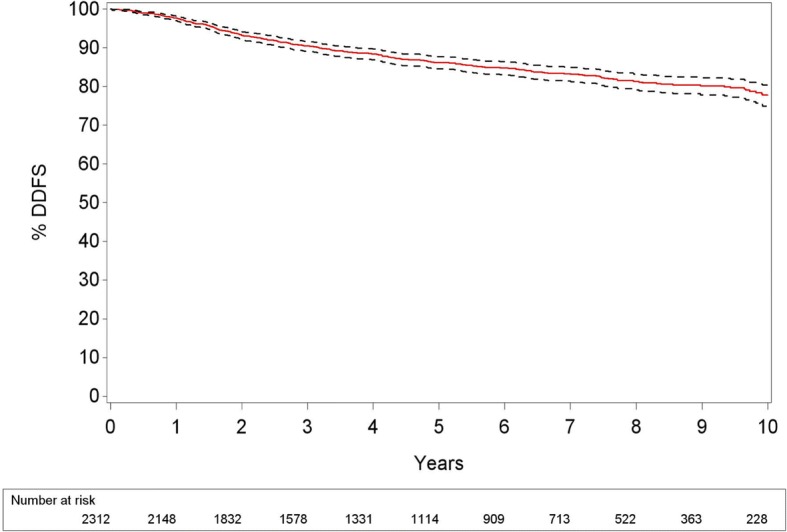
Kaplan Meier curve for distant-disease free survival +/− 95% CI

Locoregional relapse rate was low: 3/485 patients (0.6%) in the ABR group and 44/1841 (2.4%) patients in the non-ABR group. All 3 locoregional recurrences in the ABR group were rather early events after an immediate ABR: two local recurrences after 19 and 28 months and 1 nodal recurrence after 13 months. Of all the patients who had a locoregional recurrence, 28/47 (60%) also developed distant metastases during follow-up. Within this group, 15/28 patients had synchronous locoregional and distant recurrence and in 13/28 patients, the distant recurrence was detected after the locoregional recurrence. A Kaplan Meier curve for distant-disease free survival (DDFS) is shown in Fig. 3.

## Discussion

Based on the results of this study, ABR does not increase the risk of distant metastases. Moreover, the risk of metastasis after reconstruction is not time-dependent. These findings are in concordance with several other studies [25–28]. However, some of these studies focussed mainly on local recurrences [25, 26] or included also reconstructions with implants [28]. On the contrary, Isern et al. found a higher relapse rate after delayed ABR using a matched control population method [19]. In their study, prognostic or predictive factors were not considered while matching both groups. Also, no clear indications for a delayed ABR were mentioned. In a more recent study of Svee et al., only patients who underwent a delayed ABR with a DIEP flap were included [20]. In this more homogenous group of patients, an increased risk of breast cancer recurrence could not be seen.

Dillekas et al. reported a significant increase of recurrence risk during the first 2 postoperative years after a delayed breast reconstruction, including both ABR and reconstructions with implants [29]. They also revealed a different relapse pattern after surgery, depending on the type of reconstruction (implants versus ABR).

These results thus do not provide evidence for a surgery-driven escape from tumour dormancy [2–9, 15–17]. The idea of surgery-driven escape of dormancy relies on an early systemic dissemination of primary tumour cells, dormant tumour cells, at the time of diagnosis [17]. Multiple surgery-related mechanisms have been suggested to mediate this promotion of metastases [16]. As the surgical stress of an ABR is significantly higher than the surgical stress of a mastectomy alone, it was expected to see more metastases in patients who underwent an ABR compared to the patients in the non-ABR group.

Moreover, a double peaked relapse pattern of breast cancer recurrence has been described, suggesting the risk of metastasis is not constant over time [30–34]. The first peak starts 10 months after the operation and reaches its maximum approximately 18–24 months after surgery. The second peak is observed 60 months after the primary surgery. It is suggested that the first sharp peak corresponds with progressive micrometastases, present at the time of surgery, as a result of surgery-induced angiogenesis and cell division [17]. The second peak is considered the result of the natural history of breast cancer [17]. Therefor an increased risk of metastasis shortly after an ABR, compared to later follow-up times was expected. However, a relatively large increase of number of patients with metastasis for earlier follow-up times compared to later follow-up times, could not be seen in the Kaplan-Meier estimates graph (Fig. 2a). Moreover, the smoothed hazard function did not show a double peaked-pattern as well (Fig. 2b).

A potential reason for the contradictory findings in this study and the studies of Isern [19] and Dillekas [29] might be that the indications for an ABR are very strict in this centre. No immediate ABR is proposed when there is need for chemotherapy and/or radiotherapy. For a delayed ABR, the ABR is preferably performed after a disease-free interval of at least 2 years after adjuvant treatment, making remnant-circulating micrometastases in these patients less likely (potentially excluding relapsing patients from the first peak). These rather strict indications for ABR implicate that patients with an early relapse during the first 2 years after their primary surgery will not receive a delayed ABR. In order to avoid an overrepresentation of patients with an early relapse in the non-ABR group, the ABR-status is considered as a time-varying co-variate, as stated earlier. This implicates that a patient who received a delayed ABR is considered as a non-ABR patient up until the moment of the ABR. Hereby the possible overrepresentation of early relapsing patients in the non-ABR group, and thus potential bias, is avoided.

The rate of local recurrence and contralateral relapse in this cohort was too low to make any significant conclusions. Remarkably, 60% of the patients who had a locoregional recurrence also developed distant metastases. This confirms the need for extensive staging examinations and an aggressive treatment in these patients.

The strengths of this study include a large sample size of the study population, its unicentric design and the use of a prospective database. Given the nature of this study design, it has some limitations. It is a retrospective cohort study, as a randomized controlled trial is not feasible. Data on LVI were also missing in respectively 30% and 49% of the patients in this study. These values should therefore be carefully interpreted. The high amount missing data is probably due to a lack of standardization in the pathological reports in the earlier years of the cohort. Further, considering the small number of events, the study might be underpowered to detect small, but possible clinically significant differences. Lastly, it was not possible to adjust for differences in socio-economic status of the patients, as such information was not available in this database. This is considered a limitation as socioeconomic status has an impact on the prognosis and survival of breast cancer [35].

## Conclusion

In summary, this study does not show a higher risk of metastatic disease for patients with invasive breast cancer after an ABR. This data does not support a surgery-driven escape from tumour dormancy and is therefore reassuring for the selective group of patients who opt for an ABR. Prospective studies might provide better insight in the oncological safety of the ABR, in patient selection for ABR and might further explain discrepancies in the different databases.

## Abbreviations

ABR: Autologous breast reconstruction
CI: Confidence interval
DDFS: Distant-disease free survival
DIEP: Deep inferior epigastric perforator
ER: Oestrogen receptor
Her-2: Human epidermal growth factor receptor
HR: Hazard ratio
IDC: Invasive ductal carcinoma
ILC: Invasive lobular carcinoma
LVI: Lymphovascular invasion
NPI: Nottingham Prognostic Index
PR: Progesterone receptor
SGAP: Superior gluteal artery perforator
SIEA: Superficial inferior epigastric artery
TRAM: Transverse rectus abdominus myocutaneus
